# The metagenomic approach to characterization of the microbial community shift during the long-term cultivation of anammox-enriched granular sludge

**DOI:** 10.1007/s13353-017-0418-1

**Published:** 2017-12-11

**Authors:** Slawomir Ciesielski, Krzysztof Czerwionka, Dominika Sobotka, Tomasz Dulski, Jacek Makinia

**Affiliations:** 1 Department of Environmental Biotechnology, University of Warmia and Mazury in Olsztyn, Sloneczna 45G, 10-917 Olsztyn, Poland; 2 Faculty of Civil and Environmental Engineering, Gdansk University of Technology, Narutowicza 11/12, 80-233 Gdansk, Poland

**Keywords:** Anammox, Biotransformation, Deammonification, Metagenomics, Wastewater treatment

## Abstract

A metagenomic approach was used to investigate how the microbial community composition changes when an anammox-based granular sludge reactor is seeded with nitritation-anammox biomass from a wastewater treatment plant. In the seed sample, the abundance of *Candidatus* Kuenenia stuttgartiensis was similar to *Candidatus* Jettenia caeni (12.63 vs. 11.68%). This biomass was typical in terms of microbial nitrogen conversion; both ammonia (*Nitrosomonas* sp.) and nitrite (*Nitrospira* sp.) oxidizing bacteria were detected. In the lab-scale reactor, *Candidatus* Kuenenia stuttgartiensis and *Candidatus* Jettenia caeni bacteria were also present in equal proportions (18.57 vs. 20.89%). On the contrary, *Candidatus* Nitrospira defluvii bacteria were highly abundant in this reactor, but no known ammonia-oxidizing bacteria were detected. In light of recent studies showing that *Nitrospira* sp. are capable of complete nitrification, the results presented here may well indicate that both stages of nitrification in the anammox-based granular sludge reactor were performed by this bacteria.

**Electronic supplementary material:**

The online version of this article (10.1007/s13353-017-0418-1) contains supplementary material, which is available to authorized users.

## Introduction

The anaerobic ammonium oxidation (anammox) process is now a proved feasible alternative to the conventional nitrification-denitrification for nitrogen removal from anaerobic digester effluents and anaerobically treated industrial effluents. The anammox bacteria are capable of converting ammonium to nitrogen gas (N_2_) with nitrite as the final electron acceptor in the absence of dissolved oxygen (DO). Moreover, anammox does not require any organic carbon source for dissimilatory nitrogen reduction. In comparison with the conventional nitrification/denitrification process, anammox has the advantages of higher nitrogen removal rates, lower operational costs, and smaller bioreactor’s volume.

The deammonification process combines anammox with nitritation for the complete removal of ammonium from high-strength ammonium wastewater (Langone et al. 2014). In that process, ammonium oxidizing bacteria (AOB) convert approximately half of the ammonium to nitrite under DO limited conditions, and then the formed nitrite and remaining ammonium are converted to N_2_ by anammox bacteria (Pynaert et al. 2003). When organic carbon is available, heterotrophic denitrifiers may also exist in the deammonification systems and convert the nitrate produced by anammox bacteria (Kumar and Lin 2010). Because the deammonification process is strongly influenced by such factors as temperature, DO concentration, alkalinity, solids retention time, and pH (Zhang et al. 2008), the composition of microorganisms responsible for that process may be more complex and variable in comparison with the “pure” anammox process.

Although anammox-based technology has been more and more frequently used (Hu et al. 2012), the practical use of this process is still recognized as difficult mainly due to the long start-up periods which could sometimes require over one year. Such long start-up periods result from very low growth rates and biomass yields of anammox bacteria (Gutwinski et al. 2016; Ma et al. 2016). Thus, it is important to prevent the washout of anammox bacteria from the system by various techniques, including granular sludge (Gonzalez-Gil et al. 2015).

In order to shorten the long start-up periods with the anommox-based technology, anammox reactors are often inoculated with active biomass in either granular or free suspended biomass (Ni et al. 2011). Although the process of anammox start-up and enrichment has been studied (Trigo et al. 2006; López et al. 2008; Costa et al. 2014) the seeding of anaerobic anammox reactor with biomass from a nitritation/anammox process has not been fully described on the molecular level. In the present study, analysis of metagenomic data were used to investigate the seeding of a laboratory-scale sequencing batch reactor (SBR) with active AOB/anammox biomass. In order to compare the microbial communities in the seeding material and the anammox-enriched sludge in the studied SBR, 16S RNA data from next-generation sequencing (NGS) were used. In addition, the next-generation DNA sequencing was also used to compare the functional metagenomic profiles of these communities.

## Materials and methods

### Reactor set-up and operation

The biogranulation experiment was carried out in a sequencing batch reactor (SBR) with a working volume of 10 L, equipped with a control system for the measurement of DO concentrations, temperature, and pH. The SBR was operated in the cycles consisting of four phases: mixed filling (30 min), mixing and reaction (60–660 min), settling (20 min), and drawing (10 min). The whole cycle duration of one cycle varied between 2 and 12 h, depending on the actual anammox activity. The reactor was fed with synthetic medium favoring the growth of anammox bacteria (Table 1). The temperature set point was maintained at 30 °C (±1 °C), whereas pH was automatically maintained between 7.5–7.8. The SBR was operated for 52 weeks to obtain the explicit granulation effect. The values of ammonium, nitrite, and nitrate concentration in the influent and effluent are given in Table 2. The hydraulic retention time (HRT) was reduced during the SBR operation from 12 days to 8 h (Table 2). Concentrations of the nitrogen forms (NH_4_-N, NO_2_-N, NO_3_-N) were determined spectrophotometrically with cuvette tests (Hach Lange GmbH) using Xion 500 spectrophotometer (Dr Lange GmbH, Berlin, Germany), according to Standard Methods (APHA 2005).

**Table 1:** Composition of the artificial medium

Compounds	Concentration, mg L^−1^
Feeding composition	
NH_4_^+^-N	100–500
NO_2_^−^-N	100–650
KHCO_3_	1.25
CaCl_2_	1.41
KH_2_PO_4_	50.0
MgSO_4_	58.6
FeSO_4_·7H_2_O	9.08
EDTA	6.25
Trace solution* composition	
EDTA	15.0
ZnSO_4_·7H_2_O	0.43
CoCl_2_·6H_2_O	0.24
MnCl_2_·4H_2_O	0.99
CuSO_4_·5H_2_O	0.25
(NH_4_)_6_Mo_7_O_24_·4H_2_O	0.22
NiCl_2_·6H_2_O	0.20
NaSeO_4_·10H_2_O	0.20
H_3_BO_3_	0.014
NaWO_4_·2H_2_O	0.05

*- tracer solution was added in the amount of 1.25 ml L^−1^

**Table 2:** Initial and final ammonium, nitrite, and nitrate concentrations and hydraulic retention times (HRT) in the analyzed time points

Timepoint	1st day	9th week	28th week	44th week	52nd week
HRT (d)	12	1	0.66	0.33	0.33
Initial concentrations* (mg N/L)					
NH_4_-N	22.8	33.7	88.3	46.6	75.5
NO_2_-N	24.8	41.7	142.5	53.0	89.2
NO_3_-N	64.1	45.8	79.3	95.6	121.1
Final concentrations* (mg N/L)					
NH_4_-N	23.1	1.79	42.7	16.0	10.1
NO_2_-N	25.1	1.11	72.5	9.4	7.3
NO_3_-N	64.8	54.1	78.6	86.6	123.1

*- the initial and final concentrations refer to the beginning and end, respectively, of the reaction phase

### Seeding biomass

The inoculum biomass, originated from a full-scale sidestream treatment (deammonification) system in a municipal wastewater treatment plant in Zurich (Switzerland). Before the experiment began, the biomass had been stored for 32 weeks at room temperature and enriched twice a week with a mixture of NH_4_Cl and NaNO_2_. Due to the long-term storage of biomass under the substrate-limited conditions, the initial activity of anammox bacteria in the studied SBR was hardly detectable (0.016 gN gVSS^−1^ d^−1^).

### DNA extraction

Genomic DNA was extracted from 0.2 g of semidry of nitritation-anammox biomass used as inoculum (AMX_2013) and (AMX_2015) granular sludge sample collected after 52 weeks of the SBR operation. DNA was purified using FastDNA Spin Kit for soil (MP Biomedicals, USA) as per the manufacturer’s instructions. Qubit 2.0 Fluorometer (Invitrogen, USA) was used to obtain accurate DNA quantification. The purified DNA was suspended in 100 μL of deionized, DNAase free water and stored at −20 °C.

### Library preparation and Illumina sequencing

The composition of microbial communities was identified by sequencing amplicon libraries created on the base of DNA extracted from the inoculum (AMX_2013) and sample withdrawn after 51 weeks of the SBR operation (AMX_2015). For this purpose, the V3-V4 region of the 16S rRNA gene was amplified from the metagenome using Illumina recommended PCR primers. These primers were created by adding Illumina adapter overhang nucleotide sequences to the PCR primers given by Klindworth et al. (2013). Following the amplification, PCR products for each sample were purified using the Agencourt AMPure XP PCR purification system (Beckman-Coultier) and quantified using the Qubit Fluorometer (Invitrogen, Carlsbad, CA). For the whole metagenome analysis (samples AMX_2013B and AMX_2015B), libraries were prepared using Nextera® XT DNA Sample Preparation Kit (Illumina, San Diego, USA). In both approaches, the samples were dually indexed using Nextera® XT Index Kit and DNA was sequenced on an Illumina MiSeq instrument using 2 × 250 paired-end mode. For the sequencing, Miseq reagent kit v3 (Illumina, San Diego, USA) was used. All the procedures followed the manufacturers’ instructions.

### Bioinformatic analyses

Both 16S rRNA amplicons and metagenomes sequencing were performed using the Illumina approach. The sequencing results were recorded as fastq files and uploaded to the Meta Genome Rapid Annotation Subsystems Technology (MG-RAST) server for analysis (Meyer et al. 2008). Each file was subjected to quality control (QC), which included quality filtering (removing sequences with ≥5 ambiguous base pairs) and length filtering (removing sequences with a length ≥ 2 standard deviations from the mean). The automated pipeline provided by MG-RAST was also used to obtain taxonomic classification using the BLAT program referencing the SILVA database. All the identifications were made using a maximum e-value of 1e-5, a minimum identity cutoff of 90%, and a minimum alignment length of 50 bp. The functional profiling was conducted by gene annotation with SEED subsystems using a hierarchical classification at E-value cutoff of 10–5 and a minimum alignment length of 15 amino acids in MG-RAST. Visualization was carried out using KEGG mapper. Taxonomic and functional differences between metagenomes were analyzed using Statistical Analysis of Metagenomic Profiles (STAMP v. 2.1.3) (Parks and Beiko 2010). Statistically significant differences between metagenomes were identified by Fisher’s exact test combined with the Newcombe-Wilson method for calculating confidence. The Illumina metagenomic datasets are available at MG-RAST. Sequences data of the 16S rRNA gene are deposited under accession numbers 4629229.3 (AMX_2013), 4629228.3 (AMX_2015), whereas shotgun sequencing data are available under numbers: 4647330.3 (AMX_2013B), 4647329.3 (AMX_2015B).

## Results

### Reactor performance

**Fig. 1:**
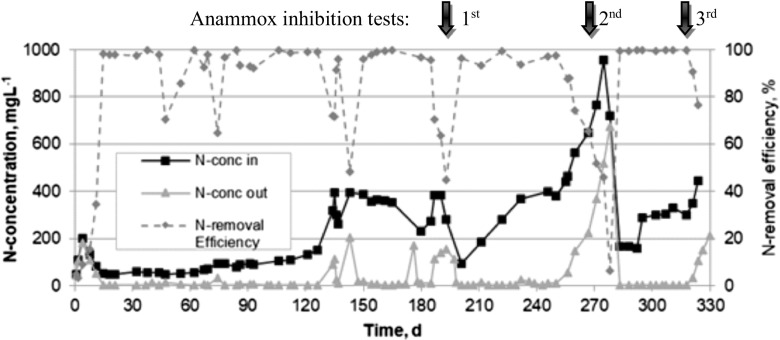
Influent and effluent nitrogen (sum of ammonia and nitrite) concentrations in the studied SBR and nitrogen removal efficiencies observed during the long-term biogranulation experiment

The anammox-enriched granular sludge was successfully formed during the long-term biogranulation experiment conducted for 51 weeks. A measurable consumption of ammonia and nitrite began to be observed in the SBR after a 2 week start-up period. Subsequently, the nitrogen load was increased by either reducing the HRT or increasing the nitrogen concentration in the synthetic feed. In the course of the biogranulation study, optimization of the operational parameters (temperature, DO, pH, and free ammonia (FA) concentration) was performed. In order to determine the impact of FA on the anammox process rate, the NLR supplied to the reactor was gradually increased during the study period from 0.1 to 8.0 kgN m^−3^d^−1^. It was found that the applied NLRs higher than 3.5 kgN m^−3^d^−1^ significantly affected the activity of anammox bacteria, which resulted in the elevated effluent concentrations of nitrogen compounds (ammonia and nitrite), e.g., on week 28 (Table 2). The nitrogen (sum of ammonium and nitrite) removal efficiency in the SBR increased from 54% on week 2 to over 98% at the end of the study (Fig. 1). Except for the periods in which the inhibitory effect of high concentration of FA on the anammox process was investigated (three intentional anammox inhibition tests on days 192, 270, and 323), the nitrogen (sum of ammonium and nitrite) removal efficiency in the studied SBR remained stably above 90%. The overall nitrogen removal rate (NRR) and specific anammox activity (SAA) reached the maximum value of 5.3 kgNm^−3^d^−1^ and 1.6 kgNkgVSS^−1^d^−1^, respectively.”

### Illumina sequencing

In order to evaluate differences between microbial community composition in the seed sample (AMX_2013) and the enriched microbial community in the lab-scale reactor (AMX_2015), the data obtained by 16S rRNA gene sequencing were compared. The characteristics of the analyzed sequences are summarized in Table 3. Taxonomic affiliations were assigned to contigs with rRNA genes based on comparison with SILVA database. Alpha diversity was much higher for the seed sample (19.24) in comparison with the sample withdrawn from the reactor (11.65). Bacteria constituted the dominant domain in both seed sample (94.2%) and enriched sample (94.3%). At the phylum level, the most abundant bacteria found in the seed sample (AMX_2013) were *Firmicutes*, *Planctomycetes*, *Proteobacteria*, *Actinobacteria*, *Chloroflexi* and *Acidobacteria*. These bacteria accounted for 21.9, 21.0, 5.9, 4.7, 2.1, and 2.0% of all the *Bacteria* reads, respectively. The most abundant phylum in sample AMX_2015 was *Planctomycetes* (50.6%), followed by *Proteobacteria* (10.4%), *Actinobacteria* (5.0%), *Bacteroidetes/Chlorobi* (1.8%), *Firmicutes* (1.6%), and *Chloroflexi* (1.2%).

**Table 3:** Metagenomic sequence statistics obtained by Illumina approach. QF – quality filtering

Sample	Total reads	Reads (post QF)	Mean sequence length (post QF) bp	Mean GC percent (post QF)	α diversity
AMX_2013	240,354	239,708	246 ± 113	56 ± 3%	19.24
AMX_2015	324,519	324,031	292 ± 62	56 ± 3%	11.65
AMX_2013_B	460,435	431,009	220 ± 50	50 ± 12%	–
AMX_2015_B	363,763	333,500	225 ± 48	49 ± 10%	–

**Fig. 2:**
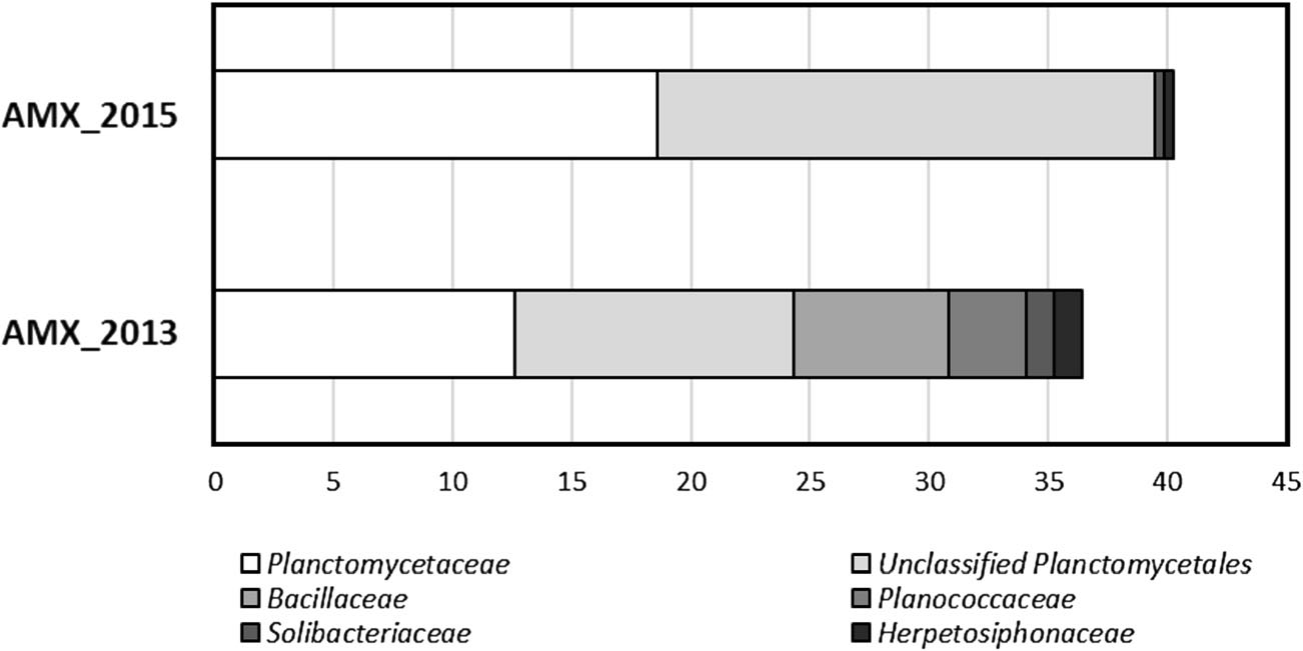
Family level affiliations assigned to contigs with 16S rRNA genes in analyzed samples. Only orders with abundance higher than 1.0% are given

**Fig. 3:**
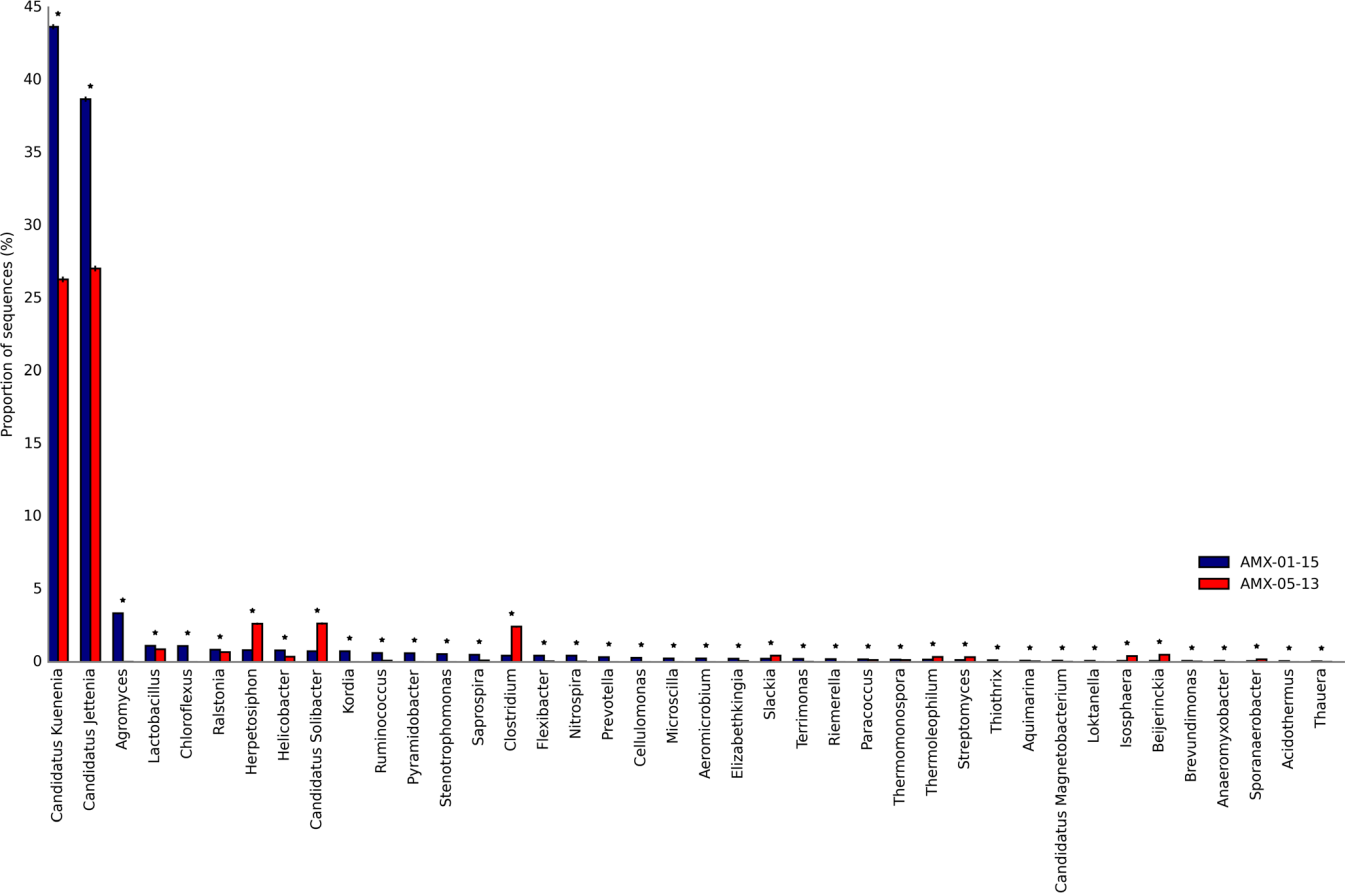
Statistically significant differences between genera in seeding sample (AMX_2013) and sample from lab-scale reactor (AMX_2015). The graphic, obtained with the STAMP software, shows the differences between the proportions of sequences in each sample with a confidence interval of 95%

At the family level, 154 and 137 families were found in AMX_2013 and AMX_2015 samples, respectively (Fig. S1). The proportions of the six major families are presented in Fig. 2. Contigs affiliated with *Planctomycetaceae* (12.63%), unclassified *Planctomycetales* (11.68%), *Bacillaceae* (6.53%), *Planococcaceae* (3.22%), *Solibacteraceae* (1.17%), *Herpetosiphonaceae* (1.17%) predominated in the seed sample (AMX_2013). In the second sample (AMX_2015), only members of *Planctomycetaceae* (18.57%) and unclassified *Planctomycetales* (20.89%) were dominant. The statistically significant differences in the abundance of all genera between AMX_2013 and AMX_2015 were shown by the STAMP software (Fig. 3). In AMX_2013, which was characterized by a higher index of biodiversity than AMX_2015, anammox bacteria *Candidatus* Kuenenia stuttgartiensis (12.63%) and *Candidatus* Jettenia caeni (11.68%) predominated. In AMX_2015, the number of anammox bacteria increased to 18.57% (*Ca*. Kuenenia stuttgartiensis) and to 20.89% (*Ca*. Jettenia caeni). Contrary to anammox bacteria, nitrifying bacteria showed differences between samples. Ammonia-oxidizing *Nitrosomonas europaea* was detected only in the seed sample (0.23%). *Candidatus* Nitrospira defluvii, contrary to *Nitrosomonas europaea*, was more abundant in the lab-scale reactor (0.2%) than in the seed sample (0.007%). The limited number of anammox bacteria in the seed sample was counterbalanced by numerous denitrifying genera such as *Bacillus*, *Sporosarcina*, *Gordonibacter*, and *Clostridium*.

**Fig. 4:**
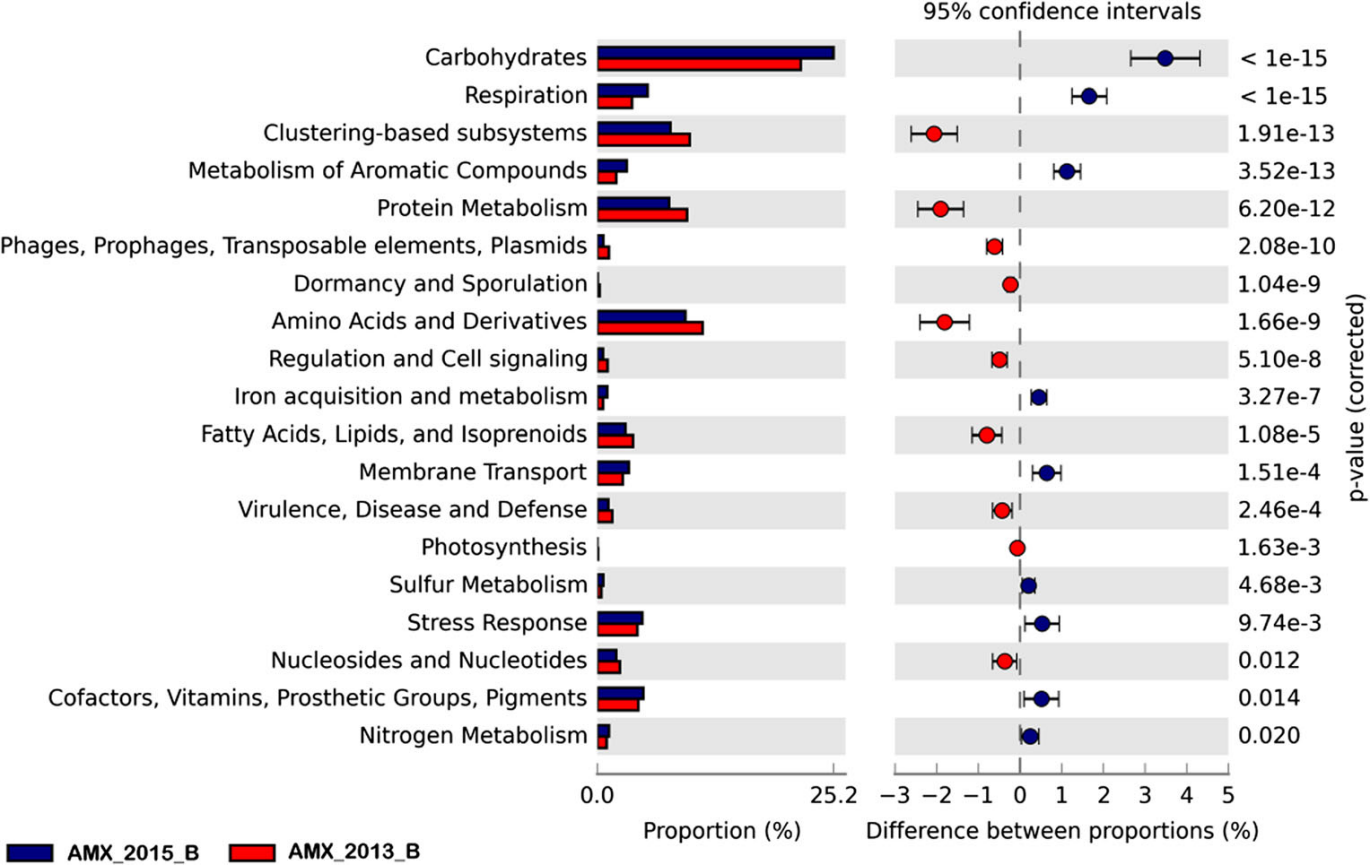
Statistically significant differences between bacterial metabolic profiles in seed sample (AMX_2013B) and sample from lab-scale reactor (AMX_2015B). The graphic obtained with the STAMP software, shows the differences between the proportions of sequences in each sample with a confidence interval of 95%

A total of 264,731 and 178,371 protein-coding regions were predicted from the AMX_2013B and AMX_2015B metagenomes, respectively. For the AMX_2013B and AMX_2015B metagenomes, 122,383 (46.2%) and 85,591 (47.9%) predicted proteins were assigned and annotated, respectively. The remaining sequences had no significant similarity to any protein in the databases. Functional analyses were performed to compare metabolism profiles of the AMX_2013B and AMX_2015B metagenomes using SEED at level 1. The pathways showing significant differences between analyzed metagenomes are shown in Fig. 4. Genes whose numbers were significantly higher in the seed sample (AMX_2013B) were related to clustering-based systems, protein metabolism, movable genetic factors (phages, prophages, transposable elements, plasmids), dormancy and sporulation, metabolism of amino acids and derivatives, regulation and cell signaling. Other genes with a significant overrepresentation in the AMX_2013B sample included those responsible for metabolism of fatty acids, lipids and isoprenoids, virulence, disease and defense, photosynthesis as well as for nucleosides and nucleotides metabolism. The AMX_2015B sample was found to have a significantly higher number of genes related to carbohydrate metabolism, respiration, metabolism of aromatic compounds and iron acquisition, membrane transport, sulfur metabolism and stress response. Moreover, in the sample from the reactor, genes related to cofactors, vitamins, prosthetic groups, pigments, and nitrogen metabolism were overrepresented.

**Fig. 5:**
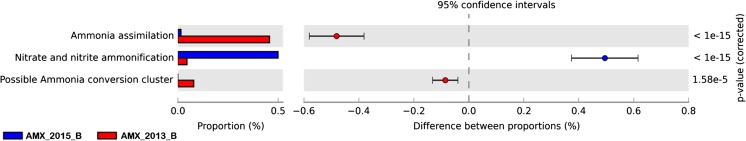
Statistically significant differences in nitrogen metabolism derived from level 3 subsystem in seeding sample (AMX_2013B) and sample from lab-scale bioreactor (AMX_2015B). The graphic obtained with the STAMP software, shows the differences between the proportions of sequences in each sample with a confidence interval of 95%

Further analyses of the nitrogen metabolism (SEED level 3) revealed that in both analyzed metagenomes genes responsible for the ammonia assimilation, denitrification, nitric oxide synthase, nitrogen fixation, and nitrosative stress were present. However, the abundance of genes belonging to ammonia assimilation, nitrate and nitrite ammonification, and possible ammonia conversion cluster was significantly different in the studied metagenomes (Fig. 5). Only one gene, coding for copper-containing nitrite reductase and representing the denitrification subsystem (*NirK*; EC 1.7.2.1), revealed a higher abundance in AMX_2013B. In the AMX_2015B metagenome, the abundance of six genes was significantly higher. Among them were three genes from the nitrate and nitrite ammonification subsystem, including respiratory nitrate reductase alpha and beta chain genes (NarG and NarH; EC 1.7.99.4) and the gene coding for nitrite reductase NAD(P)H large subunit (EC 1.7.1.4). The dissimilatory nitrite reductase subsystem was represented by the gene coding for nitrite reductase associated c-type cytochrome (*NirN*). The denitrification subsystem was represented by genes responsible for nitrous-oxide reductase (EC 1.7.99.6) and nitrous oxide reductase maturation transmembrane (*NosR*). The functional composition of both metagenomes using the similarity to a non-redundant protein database against the KEGG metabolic pathway involved in the nitrogen conversion is shown in Fig. S2.

## Discussion

The results of this study demonstrated that the abundance of *Planctomycetes* members were similar in samples withdrawn from the nitritation-anammox WWTP (the seed biomass) and samples withdrawn after the long-term cultivation in the laboratory SBR. In both samples only *Ca*. Kuenenia stuttgartiensis and *Ca*. Jettenia caeni, formerly known as planctomycete KSU-1(Ali et al. 2015), were detected. The abundance of both bacteria was 1.47 (*Ca*. Kuenenia stuttgartiensis) and 1.78 (Ca. Jettenia caeni) times higher in enriched granular sludge. Ma et al. (2017) also showed dominance of *Ca*. Kuenenia and *Ca*. Jettenia in anoxic biofilm. However, in their work *Ca*. Jettenia was much more abundant (17.83%) than *Ca*. Kuenenia (2.62%). Bae et al. (2007) examined granular anammox sludge and found that the second most common phylum was *Planctomycetes* (25%), in which the most abundant clone was also *Ca*. Jettenia caeni. Guo et al. (2016) also reported that *Planctomycetes* predominated in granular anammox sludge. Those authors found that the abundance of this phylum was 89.6% in a lab-scale reactor, however the predominant taxon was *Candidatus* Kuenenia. Chen et al. (2012) also showed that *Ca*. Kuenenia stuttgartiensis were the dominant anammox bacteria in granular sludge at high substrate concentrations. Moreover, Gonzalez-Gil et al. (2015) found that most of the bacteria in a full scale reactor belonged to *Ca*. Kuenenia stuttgartiensis as shown by MG-RAST metagenomic analysis. Although, many papers deal with anammox bacteria, our study showed for the first time, the equal abundance of *Ca*. Kuenenia and *Ca*. Jettenia. Bae et al. (2007) reported that granular sludge comprised, in addition to *Planctomycetes*, bacteria from *Proteobacteria* (47%), *Chloroflexi (18%)*, *Chlorobi* (8%), and *Acidobacteria* (2%) phyla. The structure of the microbial community in the study of Bae et al. (2007) was similar to the present study (AMX_2015 sample), even though the proportions of the specific phyli are different.

The results of the present study showed that *Ca*. Kuenenia stuttgartiensis and *Ca*. Jettenia caeni were present in similar numbers in the anammox granular sludge. One of the differences between these two bacteria is their nitrite reductase genes. *Ca*. Kuenenia stuttgartiensis possess a gene for a cytochrome *cd1*-type nitrite reductase (*nirS*), whereas *Ca*. Jettenia caeni have a copper nitrite reductase gene (*nirK*) (Hu et al. 2012). The *NirK* gene has been previously detected in denitrifiers but not in anammox bacteria other than KSU-1 (Hira et al. 2012). The presence of *nirK* in the experimental anammox reactor suggests that this nitrite reductase could play a major role in the granular anammox process.

In contrast to the typical anammox biomass, nitritation-anammox (deammonification) biomass is characterized by a high diversity of denitrifiers, showing the importance of denitrification in nitritation-anammox systems (Langone et al. 2014). In studied metagenomes (AMX_2013B and AMX_2015B), both forms of nitrite reductase, nirS and nirK, were present. Although the abundance of the *nirS* gene did not differ significantly between samples, *nirK* gene number was significantly higher in the nitritation-anammox biomass. This difference may be due to the fact that numerous denitrifiers were present in the seed sample, including *Bacillus*, *Ralstonia*, *Sporosarcina*, *Gordonibacter*, and *Clostridium*, although it is also possible that other bacteria were carrying the *nirK* gene because it is easily spread through lateral gene transfer (Heylen et al. 2006).

Until now, the full nitrification had only been described as occurring as the result of cooperation between AOB and nitrite oxdizing bacteria (NOB). However, van Kessel et al. (2015) and Daims et al. (2015) have shown that *Nitrospira* sp. is able not only to oxidize nitrite to nitrate but also to perform the first stage of nitrification, oxidizing ammonia to nitrite. In this new paradigm, *Nitrospira* sp. is not competing with anammox bacteria, but actually helping anammox bacteria by providing them with extra nitrite.

In the light of these findings, the results of the present study may indicate that, nitrification in the experimental reactor was implicitly performed by *Candidatus* Nitrospira defluvii belonging to *Nitrospira* sublineage I (Lücker et al. 2010). The evidence for these findings is that only a few (0.003%) AOB from *Nitrosomonas* genus were detected in the granular sludge sample, whereas the number of bacteria belonging to *Nitrosomonas* species was relatively high in the seed sample (0.37%). The abundance of *Ca*. Nitrospira defluvii was significantly higher in the experimental reactor in comparison with the seed sample. It is likely that, in this reactor, *Ca*. Nitrospira defluvii replaced AOB, because it is more flexible and able to survive periods of oxygen depletion (Koch et al. 2015). A similar finding was done by Bagchi et al. (2016), who noted the presence of a *Nitrospira*-like organism with a metabolic potential for complete ammonia oxidation to nitrate in a sidestream deammonification pilot reactor. The metagenomics analysis performed by the authors revealed, based on the identified ferredoxin nitrite reductase, that discovered *Nitrospira* was also taxonomically affiliated with I sublineage. The presence of a *Nitrospira*-like organism with the metabolic potential to perform the complete oxidation of ammonia to nitrate was also reported by Pinto et al. (2015). All these observations suggested, that comammox is more essential for ammonia transformation than previously thought.

Finally, the metagenomic analysis has provided interesting information about similarities in the functional traits. Focusing on the nitrogen conversion metabolic pathways, it was observed that, except for the *nirK* gene overrepresented in the seed sample, only a few genes were more abundant in the experimental reactor. Their high abundance may be related to the prevalent number of anammox bacteria in the sample from the experimental reactor. In general, metagenomic analysis gives an overview of the functional capabilities and differences between analyzed metagenomes. The most significant differences were observed for the reads related to carbohydrates, respiration, aromatic compounds and proteins metabolism, and various functions grouped in clustering-based subsystems (Fig. 4). The metabolic profile obtained for experimental (AMX_2015B) was very similar to the results reported by Gonzalez-Gil et al. (2015) for granular anammox sludge (MG-RAST accession number 4544122). The sample analyzed by Gonzalez-Gil et al. (2015) was however withdrawn from a 70 m^3^ full-scale anammox reactor. This suggests that no matter the scale, it is possible to obtain similar metabolic pathways despite differences in the microbial composition.

To sum up, the present study demonstrated that an anaerobic granular anammox reactor was successfully started up using nitritation-anammox biomass. In spite of the fact that the sample was deposited for months, it was a good source of seeding material for the anammox process. The results showed that *Ca*. *Kuenenia stuttgartiensis* could coexist with *Ca*. Jettenia caeni in nitritation-anammox biomass as well as in granular anammox biomass. The most interesting finding of this work refers to the possibility of complete nitrification by *Ca*. Nitrospira defluvii in the experiental reactor. Based on the obtained results, it could be hypothesized that *Ca*. Nitrospira defluvii were responsible for comammox in the reactor and provided the extra nitrite load for anammox bacteria.

## Electronic supplementary material

ESM 1 (DOC 1485 kb)
