# Supplementary material for: The metagenomic approach to characterization of the microbial community shift during the long-term cultivation of anammox-enriched granular sludge
Source: J Appl Genet. 2017 Dec 11;59(1):109–17. doi: 10.1007/s13353-017-0418-1 (PMC5799322; doi:10.1007/s13353-017-0418-1)
Supplement: Supplementary file 1 — (DOC 1485 kb) [file 13353_2017_418_MOESM1_ESM.doc]

Supplementary Material

**The metagenomic approach to characterization of the microbial community shift during the long-term cultivation of the anammox-enriched granular sludge**

Slawomir CIESIELSKI^1^ Krzysztof CZERWIONKA^2^, Dominika SOBOTKA^2^, Tomasz DULSKI^1^, Jacek MAKINIA^2^

^1^  Department of Environmental Biotechnology, University of Warmia and Mazury

in Olsztyn, Sloneczna 45G, 10-917 Olsztyn, Poland

^2^  Faculty of Civil and Environmental Engineering, Gdansk University of Technology,

Narutowicza 11/12, 80-233 Gdansk, Poland


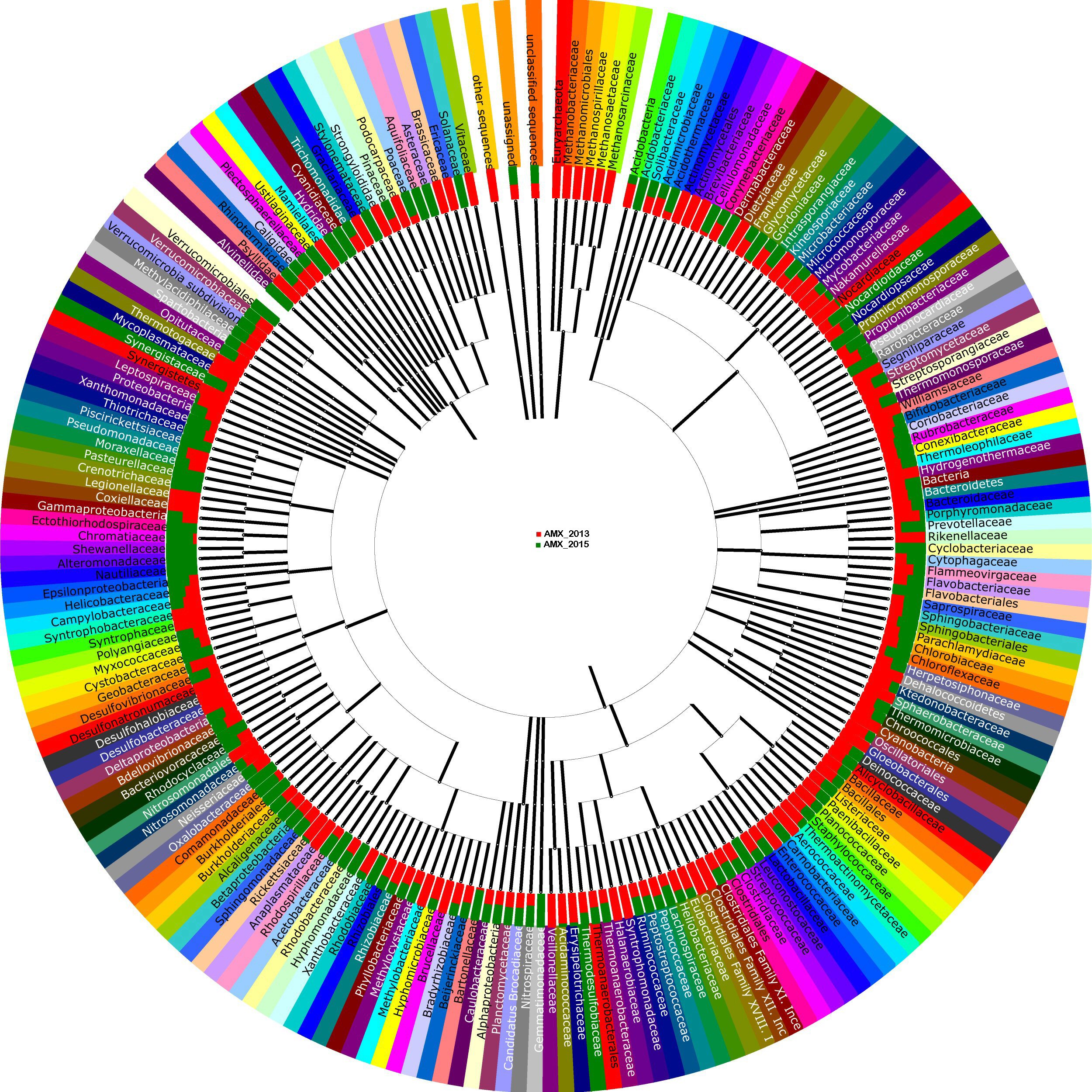


**Figure S1**. Taxonomic affiliation of metagenomic reads of the analyzed samples using MG-RAST. Bars indicate the relative abundance of the different families in the metagenomes.


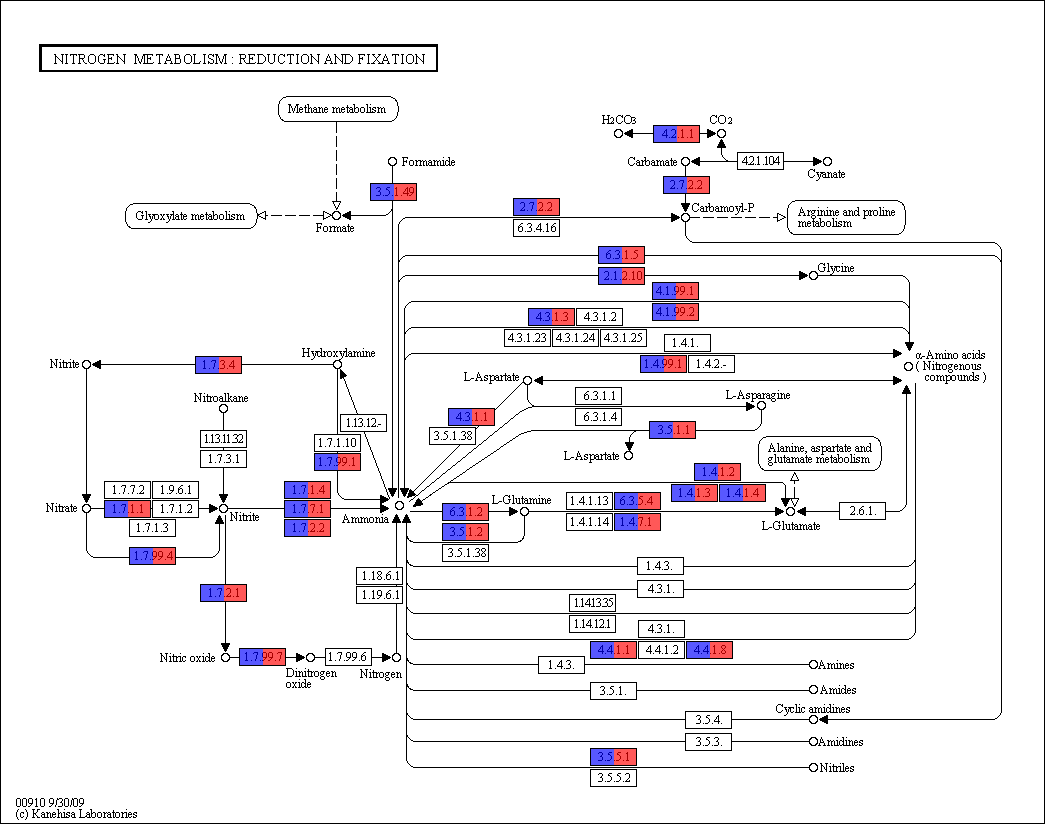


**Figure S2**. KEGG sub-pathway for nitrogen metabolism. Seed sample (AMX_2013B) - red color, sample from lab-scale reactor (AMX_2015B) - blue color.
